# Research progress on the α-synuclein-lysosome axis in Parkinson’s disease: molecular mechanisms of protein aggregation, autophagy dysfunction, and therapeutic targeting

**DOI:** 10.3389/fnins.2026.1885103

**Published:** 2026-07-27

**Authors:** Jiaojiao Fu, Chuanxi Zhu, Zhenzhen Fan

**Affiliations:** Department of Rehabilitation Medicine, The 960th Hospital of the PLA Joint Logistics Support Force, Jinan, China

**Keywords:** autophagy, lysosome, Parkinson’ s disease, protein aggregation, α-synuclein

## Abstract

Parkinson’s disease (PD) is the second most prevalent neurodegenerative disorder worldwide, characterized pathologically by the loss of dopaminergic neurons in the substantia nigra and the formation of Lewy bodies, which predominantly consist of misfolded α-synuclein (α-Syn) aggregates. Recent advances have highlighted the critical role of the interplay between α-Syn and lysosomal function, termed the α-Syn-lysosome axis, as a central mechanism underlying PD pathogenesis. This review systematically summarizes the molecular mechanisms driving α-Syn aggregation and the lysosomal dysfunction contributing to impaired autophagy-lysosome pathway (ALP) activity. We further discuss emerging therapeutic strategies targeting this axis to restore lysosomal function and mitigate α-Syn toxicity. By integrating the latest findings from molecular biology, cell biology, and preclinical studies, this article aims to elucidate the complex regulatory network of the α-Syn-lysosome axis and provide a theoretical foundation for the development of novel therapeutic interventions for PD.

## 1 Introduction

Parkinson’s disease (PD) is a common neurodegenerative disorder clinically characterized by bradykinesia, rigidity, resting tremor, and other motor symptoms, and pathologically associated with the progressive loss of dopaminergic neurons in the substantia nigra pars compacta (SNpc) (Elsworth, 2020; Hayes, 2019; Xiao et al., 2025). A defining pathological feature of PD is the presence of Lewy bodies (LBs) and Lewy neurites, which are intracellular inclusions predominantly enriched in aggregated α-synuclein (α-Syn) (Moors and Milovanovic, 2024; Shahmoradian et al., 2019). α-Syn is a 140-amino acid presynaptic protein encoded by the *SNCA* gene (Burré, 2015; Sharma and Burré, 2023). Although it is generally considered an intrinsically disordered protein in solution, it can adopt α-helical conformations upon membrane binding and is thought to participate in synaptic vesicle dynamics and neurotransmitter release (Nordengen and Morland, 2024; Ramezani et al., 2023). Under pathological conditions, α-Syn can undergo conformational conversion from soluble monomers to oligomeric intermediates and fibrillar aggregates (Cremades et al., 2012). These pathological species may impair synaptic function, disrupt organelle homeostasis, and promote neuronal toxicity, thereby contributing to PD progression (Bigi et al., 2023). This aggregation process is influenced by *SNCA* mutations or multiplications, post-translational modifications, oxidative stress, and interactions with lipids or metal ions (Flores-Leon and Outeiro, 2023; Hope et al., 2004). Familial PD cases caused by *SNCA* point mutations, duplications, or triplications further support the pathogenic importance of α-Syn dosage, conformational instability, and aggregation propensity (Jia et al., 2022; Konno et al., 2016). Furthermore, α-Syn pathology is not confined to the central nervous system but can also be detected in peripheral tissues and biofluids, including skin, blood, and extracellular vesicle-associated fractions, offering potential avenues for biomarker development while also raising challenges related to assay specificity and standardization (Gibbons et al., 2023; Mazzetti et al., 2020).

The maintenance of α-Syn proteostasis depends on the coordinated clearance of soluble, misfolded, oligomeric, and aggregated α-Syn species (Ebrahimi-Fakhari et al., 2011; Webb et al., 2003). This clearance is mediated by an interconnected proteostatic network that includes the ubiquitin–proteasome system (UPS), chaperone-mediated autophagy (CMA), macroautophagy, and lysosome-dependent degradation (Cuervo et al., 2004; Ebrahimi-Fakhari et al., 2011; Pan et al., 2008; Vogiatzi et al., 2008; Webb et al., 2003). In general, the UPS contributes to the turnover of soluble or ubiquitinated α-Syn species, whereas CMA and macroautophagy become particularly important for lysosome-dependent degradation of soluble α-Syn, oligomeric intermediates, larger aggregates, and damaged organelles (Cuervo et al., 2004; Ebrahimi-Fakhari et al., 2011; Webb et al., 2003). Lysosomes represent the terminal degradative compartment for CMA and macroautophagy and are therefore essential for maintaining proteostasis in post-mitotic neurons, which have limited regenerative capacity and are highly dependent on efficient waste clearance (Erekat, 2022; Jiao et al., 2025). In PD, lysosomal dysfunction can reduce hydrolase activity, impair autophagic flux, disturb vesicular trafficking, and weaken the degradative environment required for α-Syn clearance, thereby promoting α-Syn accumulation and toxicity (Dai et al., 2024; Teixeira et al., 2021). Several PD-associated genes converge on the endolysosomal system through distinct mechanisms rather than through a single shared defect (Follett et al., 2014; Gómez-Suaga et al., 2014; Mazzulli et al., 2011). Mutations in *GBA1* reduce glucocerebrosidase (GCase) activity and perturb glycosphingolipid metabolism; *LRRK2* mutations alter Rab-mediated vesicular trafficking, lysosomal dynamics, and selective autophagy; and *VPS35* mutations disrupt retromer-dependent endosomal sorting and lysosomal enzyme trafficking (Dai et al., 2024; Drobny et al., 2023). Among these mechanisms, *GBA1*-associated GCase deficiency provides one of the strongest links between lysosomal enzyme dysfunction and α-Syn pathology (Bae et al., 2015; Mazzulli et al., 2011). Reduced GCase activity leads to the accumulation of glycosphingolipid substrates, impairs lysosomal homeostasis, and compromises α-Syn clearance, while accumulated α-Syn can further inhibit GCase trafficking and activity, forming a bidirectional pathogenic loop (Galvagnion et al., 2022; Smith and Schapira, 2022). Importantly, reduced GCase activity has also been reported in subsets of sporadic and prodromal PD, suggesting that GCase dysfunction may represent a broader lysosomal vulnerability beyond monogenic *GBA1*-associated disease (Gegg et al., 2012; Murphy et al., 2014; Parnetti et al., 2017). In parallel, pathogenic LRRK2 activity affects endolysosomal trafficking, Rab GTPase signaling, lysosomal positioning, and mitochondrial quality control, thereby linking lysosomal dysfunction to selective autophagy and mitophagy impairment (Erekat, 2022; Jiao et al., 2025). The relationship between α-Syn aggregation and lysosomal dysfunction is bidirectional: aggregated α-Syn can disrupt vesicular trafficking, lysosomal acidification, membrane integrity, and autophagosome–lysosome fusion, whereas lysosomal deficits reduce α-Syn degradation and promote further accumulation and aggregation (Dai et al., 2024; Drobny et al., 2023). This self-reinforcing cycle provides the conceptual basis for the α-Syn–lysosome axis as a central framework for understanding proteostatic failure in PD.

Within this proteostatic network, autophagic flux represents a critical process linking α-Syn clearance, organelle quality control, and lysosomal degradation. In PD models and patient-derived systems, autophagic flux may be disrupted at multiple stages, including cargo recognition, autophagosome maturation, autophagosome–lysosome fusion, and lysosomal degradation (Pitcairn et al., 2023; Tang et al., 2021). Mechanistically, pathological α-Syn species can interfere with vesicular trafficking and SNARE/Rab-dependent fusion machinery, thereby reducing autolysosome formation and autophagic turnover (Tang et al., 2021; Zhao et al., 2021). Impaired autophagic-lysosomal degradation not only reduces intracellular α-Syn clearance but may also increase the pool of secretion-prone α-Syn species, thereby facilitating extracellular release, intercellular transfer, and pathological propagation (Birnbaum and Yue, 2025; Tang et al., 2021). Thus, defective autophagic-lysosomal flux can establish a self-amplifying cycle in which impaired degradation increases α-Syn burden, while accumulated α-Syn further compromises lysosomal function, organelle quality control, and neuronal survival (Birnbaum and Yue, 2025; Dai et al., 2024).

The mechanistic convergence of α-Syn aggregation, lysosomal dysfunction, and autophagic impairment also provides a rationale for therapeutic strategies targeting this axis. These approaches include restoration of lysosomal enzyme activity, correction of lysosomal acidification, stabilization of lysosomal membrane integrity, and enhancement of autophagic flux (Arotcarena et al., 2022; Jeon et al., 2025). For example, GCase-targeted strategies are being investigated to restore lysosomal enzyme function, particularly in *GBA1*-associated PD (Kopytova et al., 2021; Zhang et al., 2024a). Autophagy-modulating approaches may also promote α-Syn clearance by improving autophagosome maturation, autophagosome–lysosome fusion, or lysosomal degradation (Sanchez-Mirasierra et al., 2022; Sasazawa et al., 2024). In parallel, anti-α-Syn immunotherapies, small-molecule aggregation inhibitors, degradation-promoting strategies, and α-Syn-lowering approaches are being developed to reduce extracellular seeding, intercellular propagation, aggregation, or production of toxic α-Syn species, although their clinical efficacy remains to be fully established (Lang et al., 2022; Pagano et al., 2022; Volc et al., 2020). Patient-derived induced pluripotent stem cell models, dopaminergic neurons, organoids, and striatal–midbrain assembloids further provide human-relevant platforms for examining lysosomal defects, ER stress, mitochondrial dysfunction, α-Syn propagation, and therapeutic responses (Gomez-Giro et al., 2025; Tran et al., 2026). However, successful translation will require clearer target-engagement biomarkers, disease-stage-specific intervention windows, and patient stratification according to genetic background, lysosomal activity, α-Syn burden, and clinical phenotype.

Parkinson’s disease pathogenesis is closely associated with the failure of α-Syn proteostasis, in which α-Syn misfolding, impaired clearance, lysosomal dysfunction, and autophagic disruption reinforce each other. The α-Syn–lysosome axis provides an integrated framework for understanding how defective protein degradation, lipid dyshomeostasis, transcriptional dysregulation of lysosomal pathways, mitochondrial stress, and α-Syn propagation converge to promote neuronal injury. Accordingly, this review critically summarizes current evidence on α-Syn aggregation, UPS–ALP convergence, GCase dysfunction, TFEB-mediated lysosomal regulation, autophagy impairment, patient-derived disease models, and therapeutic strategies targeting this axis. By emphasizing both mechanistic advances and translational limitations, this review aims to clarify how the α-Syn–lysosome axis may inform future biomarker development, patient stratification, and disease-modifying therapeutic design in PD.1. Aggregation Mechanisms and Molecular Regulation of α-Synuclein

### 1.1 Structural features and conformational transitions of α-synuclein

Alpha-synuclein (α-Syn), encoded by the *SNCA* gene, is a 140-amino acid intrinsically disordered presynaptic protein that is implicated in synaptic vesicle clustering, SNARE-complex assembly, and the regulation of neurotransmitter release (Alam et al., 2019; Burré et al., 2014; Emanuele and Chieregatti, 2015; Stykel et al., 2021). In aqueous solution, α-Syn is largely disordered, whereas binding to acidic phospholipid membranes or synaptic vesicle-like membranes promotes the formation of amphipathic α-helical structures, which may contribute to its membrane-associated synaptic functions (Bussell et al., 2005; Davidson et al., 1998; Galvagnion, 2017; Georgieva et al., 2010; Matsuki et al., 2024). This conformational plasticity supports physiological membrane interactions but may also facilitate the transition of α-Syn toward β-sheet-rich oligomeric and fibrillar assemblies under disease-relevant conditions (Ali et al., 2026; Chen et al., 2015; Cremades et al., 2012; Davidson et al., 1998; Hjelm et al., 2025). α-Syn aggregation is generally described as a multistep process involving conformational conversion of soluble monomers into oligomeric intermediates, protofibrils, and mature amyloid fibrils (Chen et al., 2015; Cremades et al., 2012; Estaun-Panzano et al., 2023; Polinski et al., 2018). Soluble oligomeric species are often considered particularly toxic because they can disrupt membrane integrity, impair organelle function, and perturb neuronal homeostasis, although toxicity may vary according to the conformation, size, and cellular context of the α-Syn species (Alam et al., 2019; Bigi et al., 2023; Chen et al., 2015; Emin et al., 2022; Winner et al., 2011). Familial PD-associated missense mutations in the SNCA gene, including A53T, A30P, and E46K, alter α-Syn aggregation kinetics, membrane binding, or fibril architecture in mutation-specific ways (Bisi et al., 2021; Krüger et al., 1998; Maltseva et al., 2024; Polymeropoulos et al., 1997; Zarranz et al., 2004). For example, A30P reduces α-Syn binding to phospholipid vesicles, whereas E46K increases fibrillization propensity and can induce distinct fibril architectures with altered fragmentation and seeding behavior (Choi et al., 2004; Greenbaum et al., 2005; Jo et al., 2002; Zhao et al., 2020). Post-translational modifications (PTMs), including Ser129 phosphorylation, nitration, and ubiquitination, further modify α-Syn solubility, aggregation behavior, inclusion formation, and degradation (Fujiwara et al., 2002; Giasson et al., 2000; Nonaka et al., 2005; Oueslati, 2016; Pajarillo et al., 2019; Tofaris et al., 2003). Oxidative and nitrative stress, together with metal ion dyshomeostasis such as iron or copper imbalance, can further promote α-Syn modification, aggregation, propagation, and neuronal toxicity (Giasson et al., 2000; Li et al., 2020; Takahashi et al., 2002; Valensin et al., 2016). High-resolution structural studies using cryo-electron microscopy and solid-state nuclear magnetic resonance have shown that α-Syn fibrils contain β-sheet-rich amyloid cores and can adopt multiple polymorphic architectures (Guerrero-Ferreira et al., 2019; Li et al., 2018; Mehra et al., 2021; Zhao et al., 2020). Such structural polymorphism has been proposed to contribute to differences in seeding activity, cellular vulnerability, and clinicopathological heterogeneity among PD and related synucleinopathies (Guerrero-Ferreira et al., 2019; Li et al., 2018; Ohira et al., 2025). For example, the E46K mutation disrupts electrostatic interactions stabilizing the wild-type fibril fold, producing a distinct fibril architecture that is more prone to fragmentation and displays enhanced seeding capacity (Guerrero-Ferreira et al., 2019; Meade et al., 2019). Moreover, familial mutations differentially affect α-Syn’s interaction with neuronal membrane lipids such as phosphatidylcholine, sphingomyelin, and cholesterol, altering aggregation rates and cytotoxicity (Brash-Arias et al., 2024; Han et al., 2020). The interplay between α-Syn’s structural transitions, genetic mutations, PTMs, and lipid interactions orchestrates its pathological aggregation, highlighting multiple molecular checkpoints amenable to therapeutic intervention (Battis et al., 2023). Engineered binding proteins, including sequestrins, have recently been developed as experimental tools to inhibit aggregation of wild-type and mutant α-Syn by stabilizing non-amyloidogenic conformations (Tinku et al., 2025; Yoo et al., 2022). Natural compounds and small molecules, including selected flavonoids, ellagic acid, and bile acid derivatives, have also been reported to modulate α-Syn fibrillization, fibril stability, or α-Syn spreading in biochemical, cellular, or animal models; however, their clinical relevance remains to be established (Kumar et al., 2021; Ono and Yamada, 2006; Radwan et al., 2023; Zhu et al., 2004). Collectively, defining the molecular determinants of α-Syn conformational dynamics and aggregation provides a basis for understanding α-Syn-driven pathology and for developing strategies to limit toxic aggregation, propagation, and downstream neuronal injury.

### 1.2 Intercellular propagation of aggregates and prion-like behavior

**FIGURE 1:**
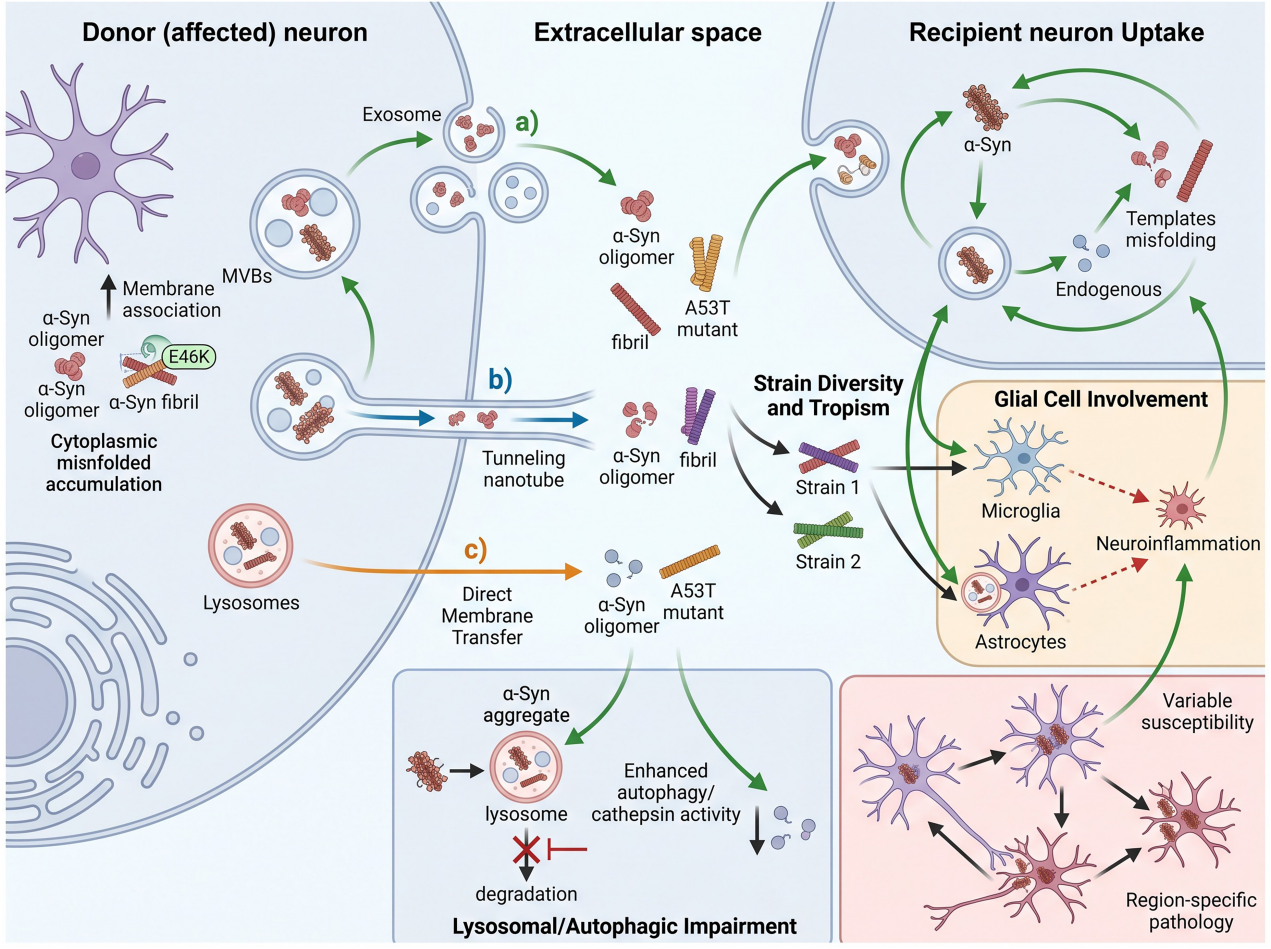
Intercellular propagation of α-synuclein and its interaction with lysosomal-autophagic dysfunction in Parkinson’s disease. Misfolded α-synuclein (α-Syn) species, including oligomers and fibrils, accumulate in affected donor neurons and can be released or transmitted to neighboring cells through multiple routes. These include (a) exosome-mediated secretion from multivesicular bodies, (b) tunneling nanotube-dependent intercellular transport, and (c) direct membrane transfer. Pathogenic α-Syn species, including mutant forms such as E46K and A53T, may escape lysosomal degradation, aggravate lysosomal-autophagic impairment, and promote further intracellular accumulation. After uptake by recipient neurons, extracellular α-Syn seeds can template the misfolding of endogenous α-Syn, thereby establishing a self-amplifying cycle of aggregation and cell-to-cell propagation. Distinct α-Syn conformational strains may exhibit differential tropism, contributing to variable neuronal susceptibility and region-specific pathology. In parallel, microglia and astrocytes participate in α-Syn handling and inflammatory responses, linking α-Syn propagation to glial activation, neuroinflammation, and progressive neurodegeneration in Parkinson’s disease.

The prion-like propagation of α-Syn aggregates has emerged as an important model for explaining the progressive anatomical spread of Lewy pathology in PD (Desplats et al., 2009; Henderson et al., 2019a; Jan et al., 2021; Luk et al., 2012; Volpicelli-Daley and Brundin, 2018). Misfolded α-Syn species, particularly oligomeric and fibrillar forms, can be released from affected neurons, taken up by neighboring cells, and seed the aggregation of endogenous α-Syn, thereby amplifying pathological α-Syn burden (Figure 1; Angot et al., 2012; Desplats et al., 2009; Luk et al., 2009; Luk et al., 2012; Tarutani and Hasegawa, 2019; Wu and Schekman, 2024). This intercellular transmission may occurs via multiple pathways, including extracellular vesicle-associated release, tunneling nanotube-mediated transfer, receptor-mediated endocytosis, and other direct uptake pathways (Figure 1; Abounit et al., 2016; Danzer et al., 2012). Extracellular vesicles, including exosomes, can carry oligomeric or aggregated α-Syn and facilitate its transfer to recipient cells (Danzer et al., 2012; Goedert et al., 2017; Minakaki et al., 2018). Disease-associated α-Syn variants, including A53T and E46K, can perturb endomembrane compartment processing and promote extracellular release and seeding of α-Syn pathology in experimental models (Figure 1; Lemkau et al., 2013; Stykel et al., 2021). The efficiency of α-Syn propagation is influenced by conformational state, because distinct α-Syn fibril polymorphs or strains can differ in seeding potency, intracellular processing, and cell-type or region-specific vulnerability (Henderson et al., 2019a; Henderson et al., 2019b; Khedmatgozar et al., 2024; Li et al., 2018; Mahul-Mellier et al., 2015; Zhang and Lingor, 2024). The Braak staging scheme describes a stereotyped caudo-rostral distribution of Lewy pathology, beginning in lower brainstem and olfactory structures and later involving cortical regions; this anatomical pattern is consistent with, although not definitive proof of, a prion-like spread model (Braak et al., 2003; Burke et al., 2008; Chen and Mor, 2023; McCann et al., 2016; Pitton Rissardo et al., 2025). After uptake by receptor-mediated or other endocytic routes, extracellular α-Syn fibrils can escape or traffic through endolysosomal compartments and template the misfolding of endogenous α-Syn, thereby amplifying intracellular aggregate formation (Figure 1; Chen K. et al., 2022; Daida et al., 2022; Mao et al., 2024; McCann et al., 2016; Wu et al., 2025). The cellular microenvironment further modulates α-Syn propagation, because astrocytes and microglia can internalize, degrade, transfer, or inflammatory amplify extracellular α-Syn species (Figure 1; Chakraborty et al., 2023; Wang et al., 2023; Xia et al., 2019; Zhang et al., 2024b). Targeting α-Syn transmission by reducing extracellular vesicle-associated release, blocking receptor-mediated uptake, or improving endolysosomal processing has therefore become an experimental therapeutic concept, although clinical efficacy remains unproven (Lang et al., 2022; Lian et al., 2026; Minakaki et al., 2018; Pagano et al., 2022). For example, improving autophagic-lysosomal degradation or cathepsin D-dependent proteolysis may reduce intracellular and internalized α-Syn seed burden, thereby limiting the pool of α-Syn species available for secretion and secondary transmission (Bae et al., 2014; Birnbaum and Yue, 2025; Jones-Tabah et al., 2024; Lee et al., 2013; Mak et al., 2010). Lipid-remodeling pathways may also influence α-Syn propagation indirectly. For example, lysophosphatidylcholine acyltransferase 1 (LPCAT1) and associated phospholipid changes have been reported to regulate α-Syn pathology and cytotoxicity in α-Syn models, suggesting that membrane lipid composition can shape aggregation-prone cellular states (Galvagnion et al., 2022; Nicholatos et al., 2022). Defining the molecular determinants of α-Syn release, uptake, endolysosomal trafficking, seeding, and glial handling may guide the development of strategies aimed at limiting pathological α-Syn spread in PD.

## 2 Lysosomal dysfunction in PD: enzymatic defects, acidification failure, and membrane instability

### 2.1 Lysosomal enzyme activity defects and *GBA1* gene mutations

**FIGURE 2:**
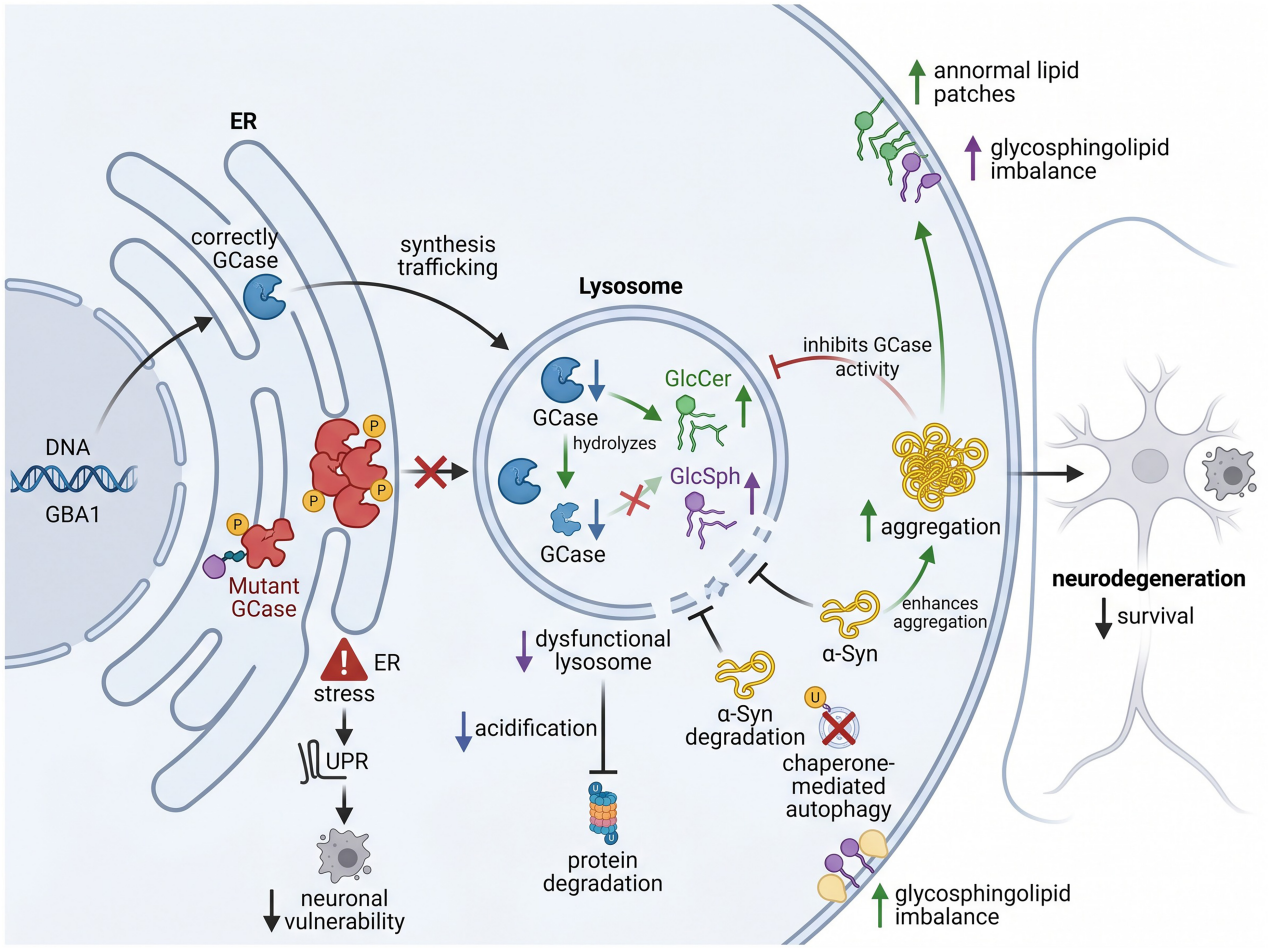
Pathogenic interaction between GBA1 mutations, GCase dysfunction, lysosomal impairment, and α-synuclein aggregation in Parkinson’s disease. GBA1 encodes the lysosomal enzyme β-glucocerebrosidase (GCase), which is synthesized in the endoplasmic reticulum (ER), correctly folded, and trafficked to lysosomes under physiological conditions. Pathogenic GBA1 mutations may impair GCase folding and trafficking, leading to ER stress, activation of the unfolded protein response, and increased neuronal vulnerability. Reduced lysosomal GCase activity disrupts the hydrolysis of glucosylceramide (GlcCer) and glucosylsphingosine (GlcSph), resulting in glycosphingolipid accumulation, abnormal lipid microdomains, lysosomal acidification defects, and impaired protein degradation. These lysosomal abnormalities compromise α-synuclein (α-Syn) clearance through autophagy-lysosomal pathways, including chaperone-mediated autophagy, thereby promoting α-Syn accumulation and aggregation. Conversely, aggregated α-Syn can further inhibit GCase activity and aggravate lysosomal dysfunction, forming a self-reinforcing pathogenic loop. This reciprocal interaction between GCase deficiency, lipid dyshomeostasis, lysosomal-autophagic failure, and α-Syn aggregation contributes to neuronal dysfunction, reduced survival, and progressive neurodegeneration in Parkinson’s disease.

The *GBA1* gene encodes the lysosomal hydrolase β-glucocerebrosidase (GCase), which hydrolyzes glucosylceramide (GlcCer) into glucose and ceramide within acidic lysosomal compartments (Mazzulli et al., 2011; Sidransky et al., 2009; Smith and Schapira, 2022; Tahanis et al., 2025). Pathogenic *GBA1* variants constitute the most common and one of the strongest genetic risk factors for PD, although the magnitude of risk varies according to variant severity, ancestry, age, and penetrance (Ran et al., 2016; Sidransky et al., 2009; Vieira and Schapira, 2022). Biallelic pathogenic *GBA1* variants cause Gaucher disease (GD), a lysosomal storage disorder, whereas heterozygous *GBA1* variants increase susceptibility to PD with incomplete and age-dependent penetrance (Chen C. et al., 2022; Omer et al., 2022; Ryan et al., 2019; Sidransky et al., 2009). Mechanistically, *GBA1* variants may reduce GCase activity, impair lysosomal delivery of GCase, or alter glycosphingolipid metabolism, leading to the accumulation of substrates such as GlcCer and glucosylsphingosine (GlcSph) (Mazzulli et al., 2011; Taguchi et al., 2017). These lipid changes can perturb lysosomal homeostasis, vesicular trafficking, and α-Syn handling (Figure 2; Dai et al., 2024; Taguchi et al., 2017). Rather than acting only as passive storage products, accumulated glycosphingolipids can alter lysosomal membrane properties, interfere with hydrolase function and trafficking, and contribute to broader ALP dysfunction (Figure 2; Mazzulli et al., 2011; Mubariz et al., 2023; Taguchi et al., 2017). As a consequence, α-Syn degradation may be compromised through multiple lysosome-dependent routes, including CMA, macroautophagy, and cathepsin-mediated proteolysis, thereby favoring α-Syn accumulation and aggregation (Figure 2; Bae et al., 2015; Mazzulli et al., 2011; Murphy et al., 2014; Sevlever et al., 2008). A bidirectional pathogenic loop may therefore develop: reduced GCase activity promotes α-Syn accumulation and oligomerization, while accumulated α-Syn can interfere with GCase trafficking, maturation, or lysosomal function, further weakening degradative capacity (Figure 2; Bae et al., 2015; Menozzi and Schapira, 2020; Murphy et al., 2014; Zheng and Fan, 2022). Patient-derived iPSC models further show that *GBA1* mutations can induce ER stress, autophagic perturbation, and increased extracellular α-Syn release in dopaminergic neurons, supporting a link between mutant GCase misfolding, proteostatic stress, and α-Syn pathology (Fernandes et al., 2016; Onal et al., 2024; Smith and Schapira, 2022; Zhang et al., 2024a). In some *GBA1* variants, misfolded mutant GCase may be retained in the ER or inefficiently trafficked to lysosomes, thereby activating UPR-related stress responses and reducing lysosomal GCase availability (Fernandes et al., 2016; Kopytova et al., 2021; Onal et al., 2024; Usenko et al., 2025; Yarkova et al., 2024). GCase deficiency also reshapes lipid homeostasis. Accumulation of GlcCer, GlcSph, and related sphingolipid abnormalities may influence α-Syn membrane binding, stabilize aggregation-prone intermediates, and impair lysosomal-autophagic degradation (Galvagnion et al., 2022; Mazzulli et al., 2011; Mu et al., 2025; Smith and Schapira, 2022; Taguchi et al., 2017; Tahanis et al., 2025). From a therapeutic perspective, GCase restoration is being explored through substrate reduction, pharmacological chaperones, small-molecule GCase activators, and gene-based approaches, although CNS delivery, target engagement, and clinical efficacy remain major challenges (Colucci et al., 2023; Menozzi and Schapira, 2020; Mullin et al., 2020; Rocha et al., 2015; Smith and Schapira, 2022; Zou et al., 2016). Pharmacological chaperones or GCase activators can increase GCase stability, trafficking, or activity in cellular and patient-derived models, and some compounds reduce α-Syn accumulation in preclinical systems; however, whether these effects translate into meaningful clinical benefit remains unresolved (Kopytova et al., 2021; Minakaki et al., 2025; Mullin et al., 2020; Ryan et al., 2019; Silveira et al., 2025; Zou et al., 2016). More recently, inhibition of cathepsin L-mediated GCase degradation has been proposed as another strategy to increase GCase protein level and activity in cellular models, but this approach remains at an early preclinical stage (Menozzi and Schapira, 2020). Overall, *GBA1*-associated PD illustrates how lysosomal enzyme deficiency, lipid dyshomeostasis, ER stress, TFEB-related transcriptional dysregulation, and α-Syn aggregation can converge on ALP failure. GCase therefore represents an important mechanistic and therapeutic entry point, but its clinical targeting will require biomarker-guided patient selection and clear evidence of target engagement.

### 2.2 Lysosomal acidification defects and V-ATPase dysfunction

**FIGURE 3:**
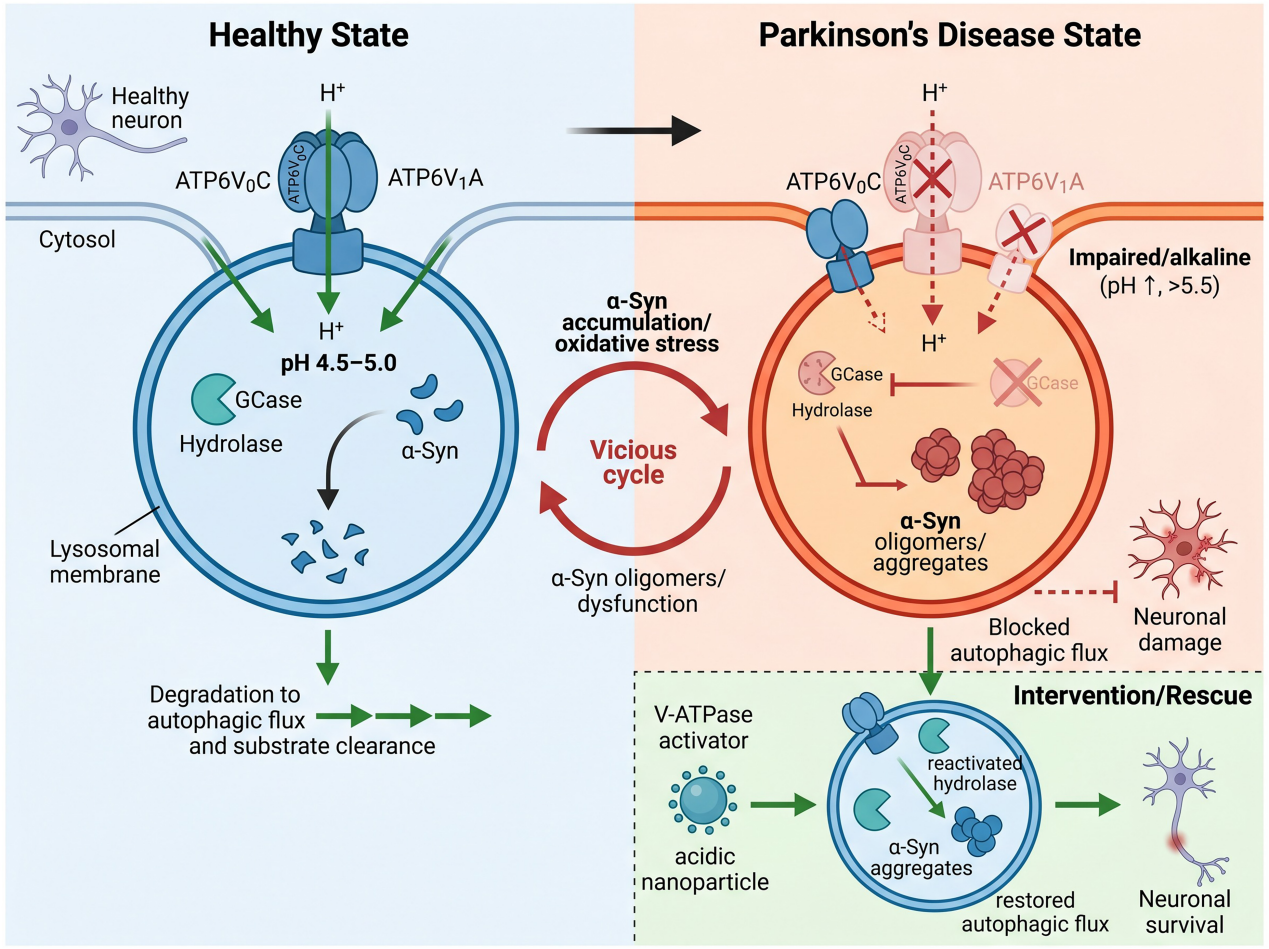
Lysosomal acidification failure and autophagic flux impairment in Parkinson’s disease. Under physiological conditions, lysosomal acidification is maintained by vacuolar ATPase subunits, including ATP6V0C and ATP6V1A, which mediate proton transport and preserve an acidic lysosomal pH of approximately 4.5–5.0. This acidic environment is essential for lysosomal hydrolase activity, α-synuclein (α-Syn) degradation, autophagic flux, and substrate clearance. In Parkinson’s disease, dysfunction or reduced activity of V-ATPase subunits impairs proton influx, resulting in lysosomal alkalinization, decreased GCase and hydrolase activity, defective protein degradation, and blocked autophagic flux. These changes promote α-Syn oligomerization and aggregation, while α-Syn accumulation and oxidative stress further aggravate lysosomal dysfunction, forming a vicious pathogenic cycle that contributes to neuronal injury. Therapeutic strategies aimed at restoring lysosomal acidity, such as V-ATPase activation or acidic nanoparticle-based rescue, may reactivate lysosomal hydrolases, enhance α-Syn aggregate clearance, restore autophagic flux, and improve neuronal survival.

Lysosomal acidification, usually maintained at an acidic luminal pH of approximately 4.5–5.0, is required for the maturation and activity of lysosomal hydrolases, including cathepsins and GCase, and thereby supports efficient degradation of α-Syn and other substrates (Figure 3; Colacurcio and Nixon, 2016; Kim et al., 2025; McGlinchey and Lee, 2015; Sevlever et al., 2008; Song et al., 2020). This acidic environment is primarily generated and maintained by the vacuolar-type H+-ATPase (V-ATPase), a multi-subunit proton pump that translocates protons into the lysosomal lumen (Colacurcio and Nixon, 2016; Falace et al., 2024; Song et al., 2020). In PD-related models, impaired V-ATPase activity or assembly can reduce proton pumping, leading to lysosomal de-acidification or alkalinization and impaired degradative capacity (Figure 3; Colacurcio and Nixon, 2016; Falace et al., 2024; Smolders and Van Broeckhoven, 2020; Song et al., 2020; Zhang S. et al., 2025). Defective acidification reduces the activity of lysosomal proteases and hydrolases, including cathepsin D, cysteine cathepsins, and GCase, thereby weakening the lysosomal degradation of α-Syn and other disease-relevant substrates (Figure 3; McGlinchey and Lee, 2015; Qiao et al., 2008; Quick et al., 2023; Sevlever et al., 2008). Oxidative stress and pathological α-Syn species may further impair lysosomal membrane function, vesicular trafficking, and acidification, thereby aggravating the reciprocal cycle between α-Syn accumulation and lysosomal dysfunction (Figure 3; Arotcarena et al., 2022; Colacurcio and Nixon, 2016; Kim et al., 2025; Song et al., 2020; Zhang S. et al., 2025). Pharmacological modulation of late endolysosomal function can also enhance α-Syn clearance. For example, activation of the endolysosomal Ca^2 +^ channel TRPML1 by ML-SA1 promotes late autophagy steps and facilitates α-Syn aggregate clearance in cellular models, although this should not be interpreted as direct V-ATPase activation (Figure 3; Kim et al., 2025; Pollmanns et al., 2022; Song et al., 2020). Acidic nanoparticles have been shown to restore lysosomal acidification and improve lysosomal function in toxin-based and α-Syn-related preclinical models, suggesting that lysosomal pH correction is a plausible experimental strategy rather than an established clinical therapy (Arotcarena et al., 2022; Bourdenx et al., 2016; Lo et al., 2026). Transcriptional regulation may further connect lysosomal acidification with broader ALP control. For instance, Nurr1 deficiency has been reported to impair autophagy-lysosomal function through GBA-dependent transcriptional regulation in PD models (Al-Nusaif et al., 2022; Cheng et al., 2025). These findings indicate that lysosomal acidification is not merely a permissive background condition but a functional requirement for hydrolase activation, α-Syn degradation, and maintenance of autophagic flux. Therefore, restoring lysosomal pH homeostasis may represent a rational therapeutic direction, but future studies are needed to define safe methods for modulating V-ATPase-related pathways, identify target-engagement biomarkers, and determine whether lysosomal re-acidification can modify disease-relevant outcomes in human PD.

### 2.3 Lysosomal membrane permeability and cathepsin release

**FIGURE 4:**
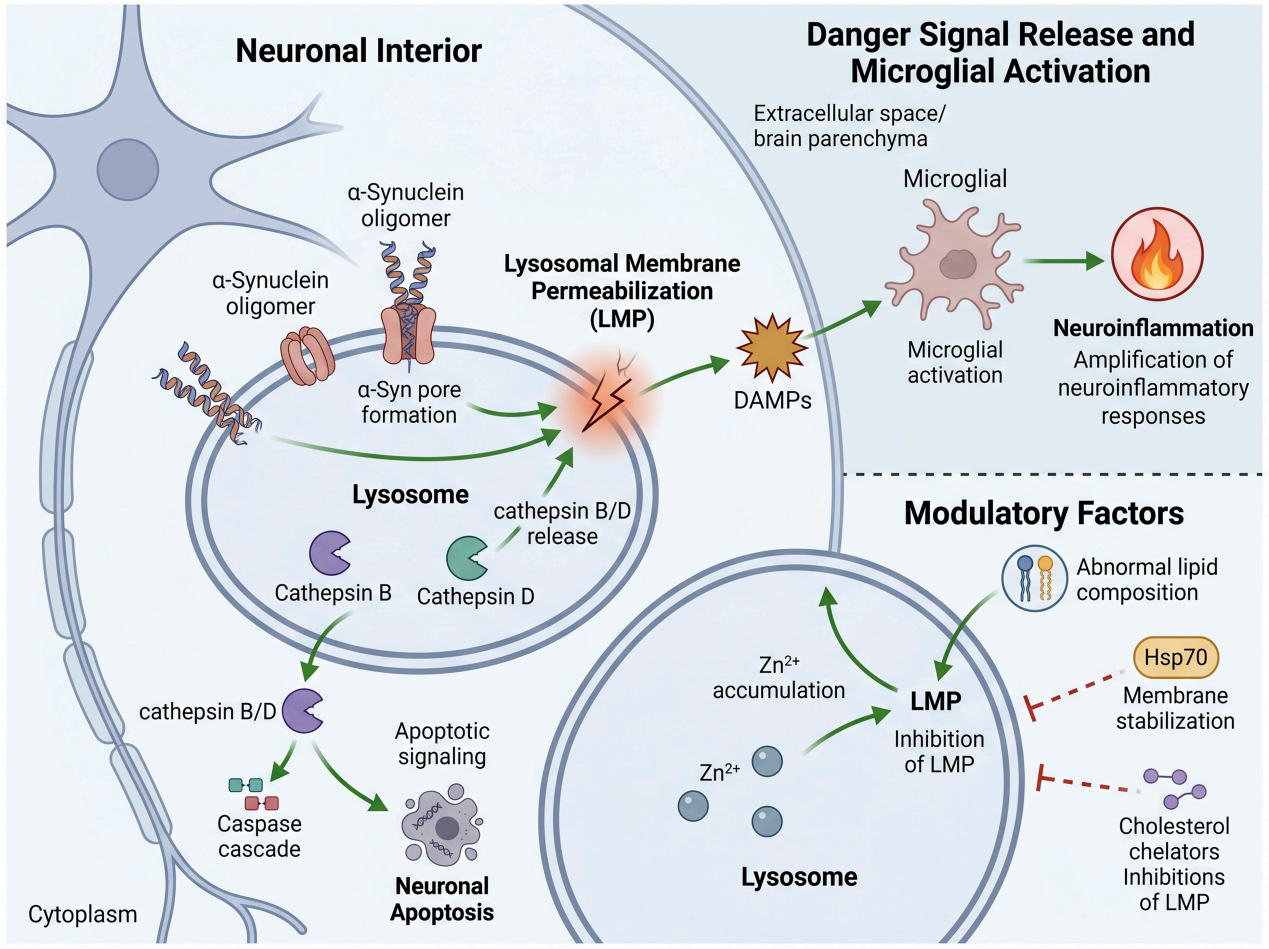
Lysosomal membrane permeabilization links α-synuclein toxicity to neuronal apoptosis and microglia-mediated neuroinflammation in Parkinson’s disease. Pathological α-synuclein (α-Syn) oligomers can interact with lysosomal membranes and induce pore formation, leading to lysosomal membrane permeabilization (LMP). Disruption of lysosomal membrane integrity promotes the release of lysosomal proteases, particularly cathepsin B and cathepsin D, into the cytoplasm, where they activate apoptotic signaling pathways and caspase cascades, ultimately contributing to neuronal apoptosis. LMP can also facilitate the release of danger-associated molecular patterns (DAMPs) into the extracellular space, thereby triggering microglial activation and amplifying neuroinflammatory responses. Several modulatory factors may influence the susceptibility of lysosomes to LMP, including abnormal lipid composition, zinc accumulation, and membrane-stabilizing mechanisms. Protective interventions such as Hsp70-mediated membrane stabilization or cholesterol-modulating strategies may inhibit LMP, preserve lysosomal integrity, attenuate neuroinflammation, and reduce α-Syn-associated neuronal damage in Parkinson’s disease.

Lysosomal membrane permeabilization (LMP) is a pathological event in which lysosomal membrane integrity is partially or extensively disrupted, allowing lysosomal contents, including cathepsins B and D, to leak into the cytosol (Figure 4; Boya and Kroemer, 2008; Dehay et al., 2010; Johansson et al., 2010; Wang et al., 2018). Once released into the cytosol, cathepsins can activate lysosome-dependent cell death pathways, promote mitochondrial dysfunction or oxidative stress, and amplify inflammatory signaling, thereby linking lysosomal injury to neuronal vulnerability and neuroinflammation (Figure 4; Bayati and McPherson, 2024; Dehay et al., 2013; Freeman et al., 2013; Wang et al., 2018). Pathological α-Syn species can compromise lysosomal integrity through at least two related mechanisms: membrane-active oligomers may form pore-like structures in lipid bilayers, whereas internalized α-Syn aggregates can induce endolysosomal or lysosomal rupture after uptake (Figure 4; Freeman et al., 2013; Kim et al., 2009; Schmidt et al., 2012). The membrane-disruptive activity of α-Syn appears to depend on oligomeric conformation, lipid composition, membrane charge, and curvature, indicating that lysosomal membrane vulnerability is shaped by both α-Syn structural state and local membrane properties (Figure 4; Freeman et al., 2013; Kim et al., 2009; Lashuel et al., 2013; Tosatto et al., 2012). Cytosolic cathepsin release can contribute to lysosome-dependent cell death, mitochondrial injury, and ROS generation; in α-Syn-treated cells, lysosomal rupture has also been linked to cathepsin B-dependent oxidative stress and inflammasome activation, providing a potential bridge between lysosomal damage and neuroinflammation (Freeman et al., 2013; Wang et al., 2018). Experimental studies suggest that lysosomal membrane stability can be influenced by chaperone systems, membrane lipid composition, oxidative stress, and metal ion homeostasis (Eriksson et al., 2017; Johansson et al., 2010; Kirkegaard et al., 2010; Nylandsted et al., 2004). For example, Hsp70 can stabilize lysosomal membranes and inhibit LMP, partly by interacting with the endolysosomal lipid BMP (Kirkegaard et al., 2010; Nylandsted et al., 2004). In addition, cholesterol and other membrane lipids may influence the susceptibility of lysosomes to α-Syn-induced membrane injury in a context-dependent manner (Eriksson et al., 2017). Zinc dyshomeostasis may also contribute to lysosomal destabilization, because excessive intracellular or lysosomal zinc loading has been linked to LMP, oxidative injury, and neuronal death in experimental systems (Figure 4; Lee et al., 2026). Interventions aimed at preserving lysosomal membrane integrity, reducing α-Syn-induced membrane injury, or limiting harmful cathepsin leakage therefore represent experimental therapeutic directions, but their disease-modifying relevance in human PD remains uncertain (Eriksson et al., 2017; Freeman et al., 2013; Kirkegaard et al., 2010; Nylandsted et al., 2004). Overall, LMP provides a mechanistic link between lysosomal dysfunction, α-Syn toxicity, cathepsin-dependent stress responses, and neuroinflammatory signaling, although the extent to which this pathway drives human PD progression requires further validation.

## 3 Autophagy impairment and mitochondrial crosstalk in α-synuclein proteostasis

### 3.1 Macroautophagy blockade and α-Syn accumulation

**FIGURE 5:**
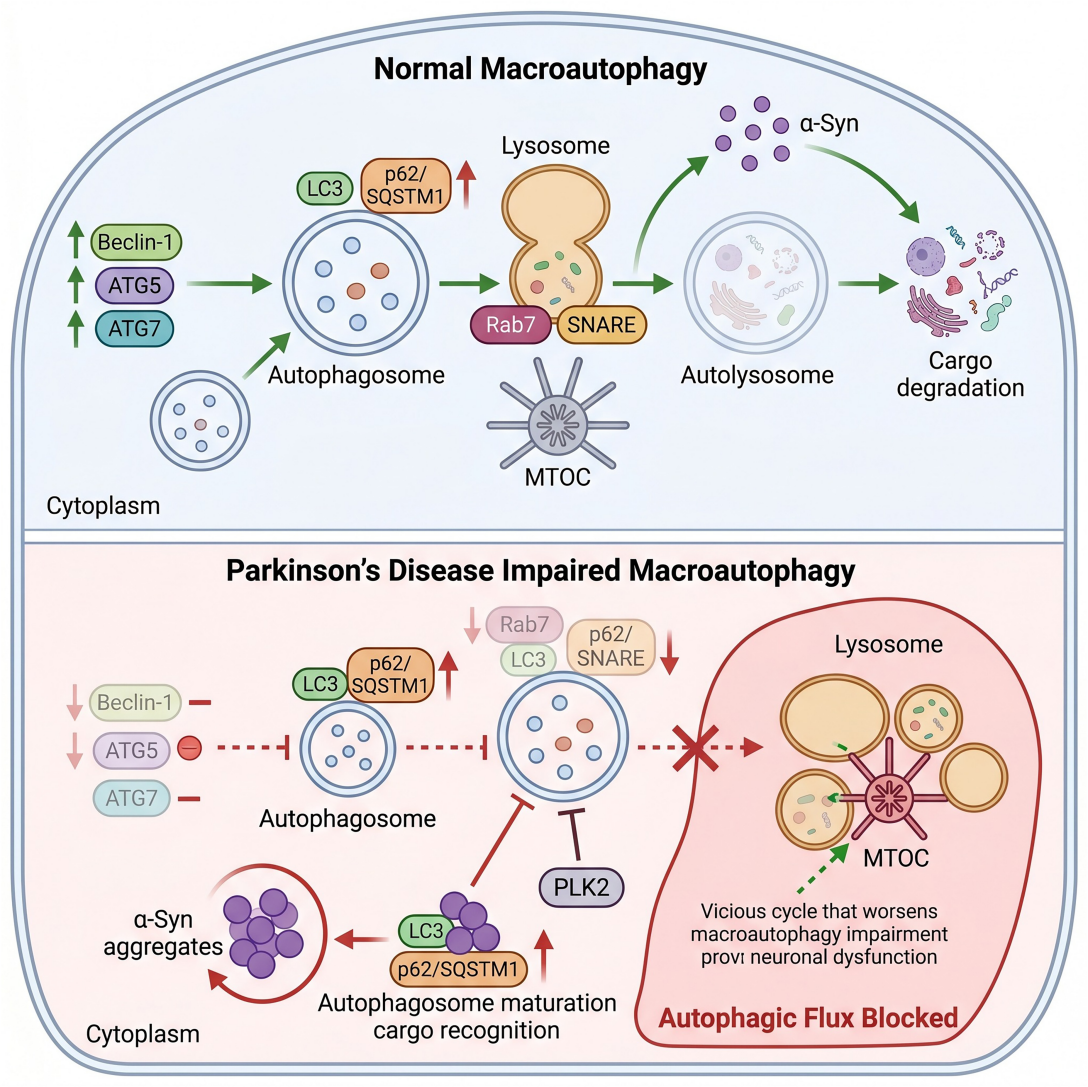
Macroautophagy impairment and α-synuclein accumulation in Parkinson’s disease. Under physiological conditions, macroautophagy maintains neuronal proteostasis through sequential steps including autophagosome initiation, cargo recognition, autophagosome maturation, vesicular transport, autophagosome–lysosome fusion, autolysosome formation, and lysosomal degradation. Core autophagy-related proteins and receptors, such as Beclin 1, ATG5, ATG7, LC3, and p62/SQSTM1, participate in autophagosome formation and selective cargo handling, whereas Rab7, SNARE proteins, and microtubule-organizing center (MTOC)-associated lysosomal positioning support autophagosome–lysosome fusion and autolysosomal degradation. In Parkinson’s disease and related experimental models, disruption of these steps may impair autophagic flux and reduce the clearance of α-synuclein (α-Syn)-containing aggregates. Accumulated α-Syn can further interfere with autophagosome maturation, selective autophagy receptor function, SNARE/Rab-dependent fusion, and lysosomal degradation, thereby reinforcing macroautophagy dysfunction. PLK2-related regulation may also influence α-Syn turnover in a context-dependent manner. Overall, impaired macroautophagic flux provides a mechanistic link between defective proteostasis, α-Syn accumulation, and neuronal vulnerability in Parkinson’s disease.

Macroautophagy is a major lysosome-dependent degradation pathway that removes long-lived proteins, protein aggregates, and damaged organelles, including α-Syn-containing aggregates under pathological conditions (Birnbaum and Yue, 2025; Spencer et al., 2009; Webb et al., 2003; Winslow et al., 2010). This process requires the formation of double-membrane autophagosomes, transport of these vesicles along the cytoskeleton, and their fusion with lysosomes to form degradative autolysosomes (Figure 5; Birnbaum and Yue, 2025; Pitcairn et al., 2023; Tang et al., 2021; Webb et al., 2003). In PD and related synucleinopathy models, impairment of macroautophagy has been implicated as an important contributor to α-Syn accumulation, impaired proteostasis, and neuronal vulnerability (Figure 5; Huang et al., 2012; Spencer et al., 2009; Tang et al., 2021; Webb et al., 2003; Winslow et al., 2010). At the level of autophagosome biogenesis, α-Syn overexpression can impair early macroautophagy by interfering with Rab1a-dependent trafficking and autophagosome formation, whereas Beclin 1-mediated autophagy activation has been shown to reduce α-Syn pathology in experimental models (Figure 5; Huang et al., 2012; Miki et al., 2016; Spencer et al., 2009; Winslow et al., 2010). Defects in autophagosome–lysosome fusion represent another important level of flux impairment. Rab7 promotes α-Syn aggregate clearance by facilitating autophagosome–lysosome fusion, whereas α-Syn can impair SNARE-dependent fusion through mechanisms involving SNAP29 and YKT6 (Figure 5; Birnbaum and Yue, 2025; Cuddy et al., 2019; Dinter et al., 2016; Pitcairn et al., 2023; Tang et al., 2021). Such fusion defects may block autophagic flux, causing autophagosomes and sequestered cargoes, including α-Syn aggregates, to accumulate rather than proceed to lysosomal degradation (Figure 5). Selective autophagy receptors also participate in α-Syn handling. p62/SQSTM1 can mediate the autophagic targeting of Lewy body-like α-Syn inclusions, while impairment of p62-dependent flux may favor α-Syn aggregation, secretion, or spread (Figure 5; Oh et al., 2022; Watanabe et al., 2012; Winslow et al., 2010; Xu et al., 2025). These findings support a feed-forward relationship in which impaired macroautophagy increases α-Syn burden, while accumulated α-Syn further disrupts autophagosome formation, maturation, fusion, or lysosomal degradation (Figure 5). Human evidence is generally consistent with altered autophagy-lysosomal regulation, but interpretation of static markers requires caution. Postmortem studies have reported changes in LC3, LAMP1, ULK1, and p62 in PD-related brain regions, including G2019S LRRK2-associated PD; however, LC3 or p62 levels alone cannot distinguish increased autophagosome formation from impaired downstream degradation (Figure 5; Huang et al., 2012; Mamais et al., 2018; Miki et al., 2016; Pitcairn et al., 2023). Experimental modulation of macroautophagy supports the concept that restoring balanced autophagic flux can reduce α-Syn burden in cellular and animal models, but therapeutic translation will require strategies that enhance clearance without causing excessive autophagy activation or lysosomal stress (Date et al., 2024; Oueslati et al., 2013; Pollmanns et al., 2022; Spencer et al., 2009). Recent work further suggests that lysosomal positioning can influence autophagic flux. Clustering of lysosomes near the microtubule-organizing center can facilitate autophagosome–lysosome fusion, and lysosome-clustering compounds have been reported to enhance insoluble α-Syn degradation in cellular PD models (Figure 5 Lee et al., 2024; Sasazawa et al., 2024). The role of PLK2 in α-Syn turnover appears context-dependent. Earlier studies suggested that PLK2 can promote selective autophagic degradation of α-Syn, whereas recent work indicates that excessive or disease-context-dependent PLK2 activity may disrupt autophagic flux and promote α-Syn pathology (Dahmene et al., 2017; Oueslati et al., 2013; Zhang C. et al., 2025). Collectively, available evidence indicates that α-Syn proteostasis can be disrupted at multiple steps of macroautophagy, including autophagosome biogenesis, cargo recognition, vesicular transport, autophagosome–lysosome fusion, and lysosomal degradation. This multilevel impairment provides a plausible mechanism by which α-Syn accumulation and autophagic failure reinforce each other during PD progression.

### 3.2 Selective impairment of chaperone-mediated autophagy

Chaperone-mediated autophagy (CMA) is a selective lysosomal degradation pathway that recognizes soluble cytosolic proteins containing KFERQ-like motifs and translocates them directly across the lysosomal membrane for degradation; soluble α-Syn is one such CMA substrate (Chiang et al., 1989; Cuervo and Dice, 1996; Cuervo and Wong, 2014; Cuervo et al., 2004; Huang and Wang, 2025). In this pathway, cytosolic Hsc70 recognizes the CMA-targeting motif and delivers the substrate to lysosomal-associated membrane protein 2A (LAMP2A), whose assembly into a translocation complex enables substrate import and lysosomal degradation (Chiang et al., 1989; Cuervo and Dice, 1996; Wang and Lu, 2023; Yang et al., 2016). In PD, CMA dysfunction is considered an important contributor to α-Syn proteostatic failure, particularly because impaired CMA favors the accumulation of soluble or modified α-Syn species that may subsequently enter aggregation pathways (Alvarez-Erviti et al., 2010; Cuervo et al., 2004; Martinez-Vicente et al., 2008; Murphy et al., 2015). Pathogenic α-Syn mutants, including A30P and A53T, as well as dopamine-modified α-Syn, can bind abnormally to the CMA receptor LAMP2A but fail to translocate efficiently into the lysosomal lumen, thereby blocking CMA-mediated degradation of α-Syn and other substrates (Cuervo et al., 2004; Lopes da Fonseca et al., 2015; Martinez-Vicente et al., 2008; Xilouri et al., 2009). CMA blockade can increase cytosolic α-Syn levels and may induce compensatory macroautophagy; however, when macroautophagic flux is also inefficient, this compensation may be insufficient to maintain homeostasis (Erekat, 2022; Martinez-Vicente et al., 2008; Massey et al., 2006; Winslow et al., 2010). Experimental enhancement of CMA, particularly by increasing LAMP2A expression, can promote α-Syn clearance and reduce α-Syn-associated toxicity in cellular and animal models (Issa et al., 2018; Xilouri et al., 2013a; Xilouri et al., 2013b). NRF2 signaling has also been linked to CMA regulation through transcriptional control of LAMP2A, and recent work suggests that apigenin may promote α-Syn clearance by activating NRF2-associated CMA pathways in experimental models (Huang et al., 2025; Pajares et al., 2018). Beyond neurons, autophagic pathways also contribute to the handling of α-Syn in glial cells; in multiple system atrophy models, CMA and macroautophagy have been implicated in the clearance of oligodendroglial α-Syn and TPPP/p25A, supporting the broader relevance of lysosomal proteostasis across synucleinopathies (Mavroeidi et al., 2022). Thus, CMA and α-Syn influence each other bidirectionally: mutant, dopamine-modified, or excessive α-Syn can impair CMA, whereas CMA dysfunction promotes the accumulation of soluble α-Syn species that may later feed into oligomerization and aggregation (Alvarez-Erviti et al., 2010; Cuervo et al., 2004; Martinez-Vicente et al., 2008; Murphy et al., 2015). Restoring CMA function, for example by increasing LAMP2A-dependent substrate translocation or enhancing CMA-related stress-response pathways, represents a rational experimental strategy for reducing α-Syn burden; however, its disease-modifying relevance in human PD remains to be established (Huang et al., 2025; Pajares et al., 2018; Xilouri et al., 2013b). Overall, CMA represents an important component of α-Syn proteostasis, particularly for soluble and modified α-Syn species, and its impairment may cooperate with macroautophagy and lysosomal defects to promote proteostatic stress in PD.

### 3.3 Interaction between autophagy dysfunction and mitochondrial impairment

**FIGURE 6:**
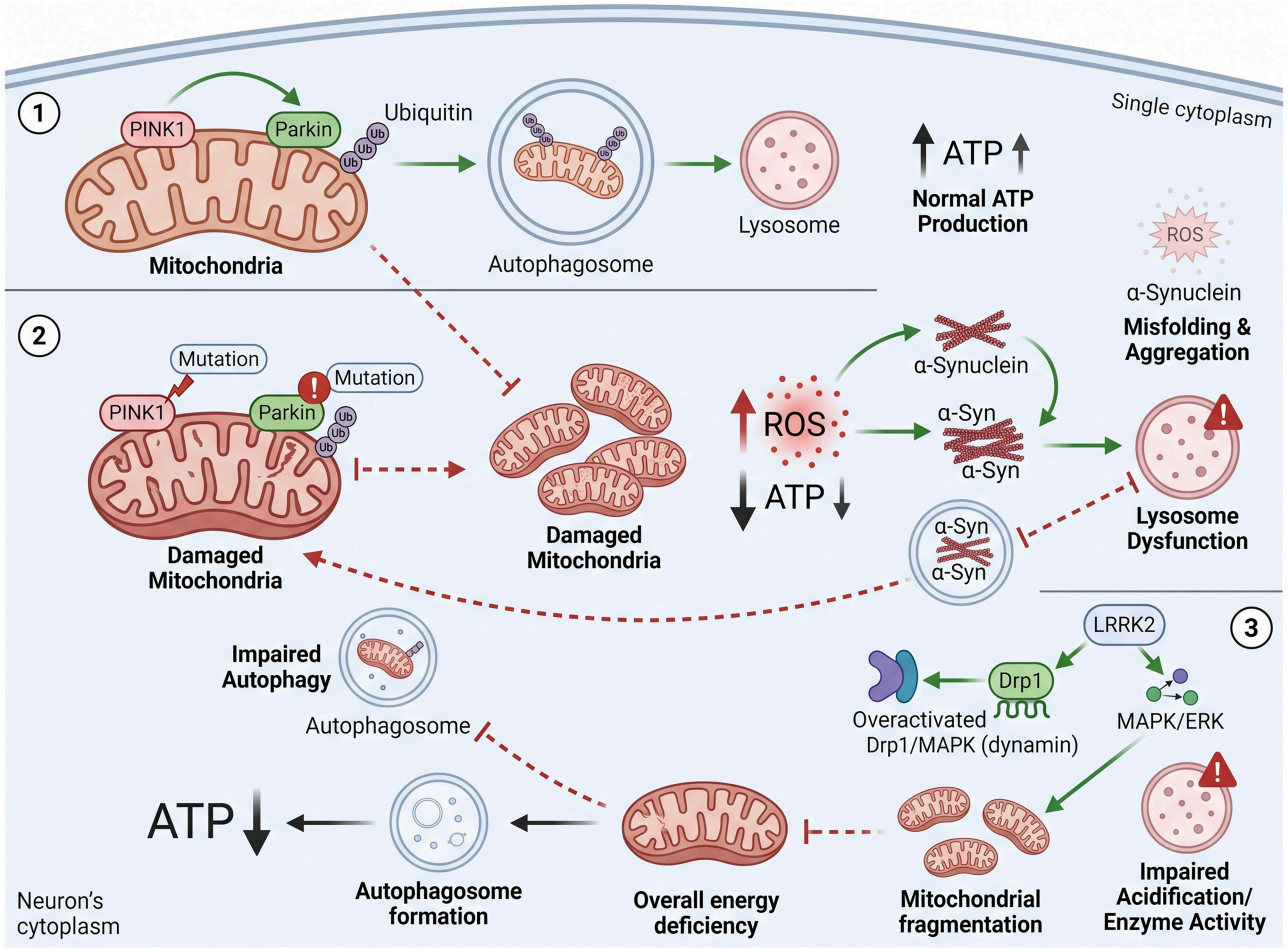
Crosstalk among mitochondrial dysfunction, impaired mitophagy, lysosomal failure, and α-synuclein pathology in Parkinson’s disease. Under physiological conditions, damaged or depolarized mitochondria are recognized by the PINK1–Parkin pathway, ubiquitinated, engulfed by autophagosomes, and delivered to lysosomes for degradation, thereby maintaining mitochondrial quality control, ATP production, calcium buffering, and redox balance. In Parkinson’s disease-related contexts, mutations or dysfunction of *PINK1*, *PRKN*, or *LRRK2* may impair mitophagy and mitochondrial dynamics, leading to the accumulation of damaged mitochondria, increased reactive oxygen species (ROS), reduced ATP production, and heightened neuronal vulnerability. Mitochondrial stress can promote oxidative or nitrative modification of α-synuclein (α-Syn), thereby favoring α-Syn misfolding, aggregation, and propagation. Conversely, excess or aggregated α-Syn may further disrupt mitochondrial dynamics, Miro turnover, PINK1–PRKN-mediated mitophagic flux, autophagosome–lysosome fusion, and lysosomal degradation. LRRK2-related alterations in DNM1L/Drp1 regulation and MAPK/ERK-related stress responses may further contribute to mitochondrial fragmentation and defective clearance. Together, these interactions form a self-reinforcing network linking mitochondrial quality-control failure, oxidative stress, lysosomal-autophagic impairment, and α-Syn proteotoxicity in Parkinson’s disease.

Autophagy dysfunction and mitochondrial impairment form an important pathogenic interface in PD, because defective organelle clearance and mitochondrial stress can reinforce each other during neuronal injury (Gao et al., 2017; Lizama and Chu, 2021; Lurette et al., 2023; Matsuda et al., 2010; Narendra et al., 2008; Shaltouki et al., 2018). Mitophagy is a selective form of macroautophagy that removes damaged or depolarized mitochondria, thereby helping to maintain mitochondrial quality control, ATP production, calcium buffering, and redox balance (Figure 6; Liu et al., 2019; Matsuda et al., 2010; Narendra et al., 2008; Narendra et al., 2010). In PD-relevant models, defective mitophagy can allow dysfunctional mitochondria to persist, resulting in impaired membrane potential, reduced ATP production, increased reactive oxygen species (ROS), and heightened vulnerability of dopaminergic neurons (Figure 6; Matsuda et al., 2010; Narendra et al., 2008; Narendra et al., 2010; Shaltouki et al., 2018; Williamson et al., 2023). Mutations in PD-associated genes such as *PINK1* and *PRKN* this pathway by disrupting the PINK1–Parkin ubiquitin signaling cascade, in which PINK1 accumulates on damaged mitochondria, recruits and activates Parkin, and promotes ubiquitin-dependent labeling of mitochondria for autophagic clearance (Figure 6; Gao et al., 2017; Kane et al., 2014; Matsuda et al., 2010; Narendra et al., 2008; Narendra et al., 2010; Shen and Dettmer, 2024). Mitophagy deficiency may also favor α-Syn pathology by increasing mitochondrial ROS and oxidative or nitrative stress, which can promote α-Syn modification, aggregation, and spread under disease-relevant conditions (Figure 6; Hsu et al., 2000; La Vitola et al., 2024; Parihar et al., 2009; Shen and Dettmer, 2024). Conversely, excess or aggregated α-Syn can interfere with mitochondrial quality control by affecting mitochondrial dynamics, Miro turnover, PINK1–PRKN-mediated mitophagic flux, and downstream lysosomal degradation, thereby reinforcing mitochondrial and proteostatic stress (Figure 6; Guerra et al., 2019; Hou et al., 2023; Lurette et al., 2023; Shaltouki et al., 2018; Shen and Dettmer, 2024). Experimental interventions aimed at improving mitochondrial quality control also support this concept. Urolithin A has been reported to protect dopaminergic neurons in experimental PD models by improving mitochondrial homeostasis, whereas the NAD^+^ precursor nicotinamide riboside rescues mitochondrial defects and neuronal loss in patient-derived neuronal and fly models; however, these findings remain preclinical or early translational rather than definitive evidence of disease modification (Brakedal et al., 2022; Liu et al., 2022; Schöndorf et al., 2018). Mutant LRRK2 provides another link between mitochondrial dynamics and mitophagy. In cellular and animal models, pathogenic LRRK2 variants have been associated with mitochondrial fragmentation, altered DNM1L/Drp1 regulation, impaired MAPK/ERK-related stress responses, and reduced mitophagic clearance (Figure 6; Liu et al., 2021; Wang et al., 2012; Wauters et al., 2020; Williamson et al., 2023). Because mitophagy ultimately depends on lysosomal degradation, defects in lysosomal acidification, hydrolase activity, or autophagosome–lysosome fusion can prevent damaged mitochondria from being fully cleared, even when upstream mitochondrial labeling occurs (Tang et al., 2021; Zhang et al., 2023; Zhang S. et al., 2025). Mitochondrial energy failure may in turn weaken ATP-dependent processes required for autophagosome formation, cytoskeletal transport, vesicle fusion, and lysosomal maintenance, thereby making autophagic clearance less efficient under sustained stress (Figure 6; Lizama and Chu, 2021; Luo et al., 2022; Lurette et al., 2023; Pitcairn et al., 2023; Shaltouki et al., 2018). Collectively, these findings support a self-reinforcing network in which defective autophagy and mitophagy permit damaged mitochondria to accumulate, while mitochondrial stress increases oxidative injury, α-Syn aggregation, and lysosomal burden. Therapeutic approaches that enhance mitochondrial quality control while preserving lysosomal degradative capacity may therefore represent rational experimental directions, but their efficacy, safety, and patient-selection requirements need to be established in human PD.

## 4 Therapeutic strategies targeting the α-Syn-lysosome axis

### 4.1 Drug development to enhance lysosomal function

Enhancing lysosomal function has become a rational therapeutic strategy for improving α-Syn clearance and reducing lysosomal stress in PD, although most approaches remain experimental or require further clinical validation (Arotcarena et al., 2022; Dai et al., 2024; Jiao et al., 2025; Mazzulli et al., 2011; Mullin et al., 2020). One key lysosomal enzyme implicated in α-Syn degradation is glucocerebrosidase (GCase), encoded by the *GBA1* gene (Chatterjee and Krainc, 2023; Kuo et al., 2022). Pathogenic *GBA1* variants, and in some cases reduced GCase activity in sporadic PD, can impair lysosomal hydrolysis and favor α-Syn accumulation (Bae et al., 2015; Kumar et al., 2023; Mazzulli et al., 2011; Murphy et al., 2014; Onal et al., 2024). Small molecule GCase modulators, including pharmacological chaperones and noninhibitory GCase activators, aim to improve GCase folding, lysosomal trafficking, stability, or enzymatic activity (Khanna et al., 2010; Mullin et al., 2020). Ambroxol is the most clinically advanced example in PD, whereas isofagomine has mainly provided proof-of-concept evidence for GCase chaperoning in Gaucher-related models (Aflaki et al., 2016; Sun et al., 2012). Ambroxol has shown target engagement in preclinical and clinical studies by increasing GCase-related measures, and experimental studies suggest that it may reduce α-Syn-related pathology in selected models; however, its disease-modifying efficacy in PD has not yet been established (Migdalska-Richards et al., 2016; Mullin et al., 2020; Prieto Huarcaya et al., 2022; Silveira et al., 2025). Ambroxol has entered clinical testing in PD and PD dementia, supporting its translational relevance, but larger controlled trials are required to determine clinical efficacy (Colucci et al., 2023; Mullin et al., 2020; Silveira et al., 2025).

Noninhibitory GCase chaperones or activators, such as NCGC607, have also been reported to reduce α-Syn and glycolipid levels in iPSC-derived dopaminergic neurons from patients with Gaucher disease and parkinsonism, supporting further development of GCase-targeted strategies (Aflaki et al., 2016). Beyond GCase, lysosomal acidification remains essential for hydrolase maturation and activity, and therefore represents another therapeutic entry point for restoring lysosomal degradation (Arotcarena et al., 2022; Bourdenx et al., 2016; Colacurcio and Nixon, 2016; Lo and Zeng, 2023; Xue et al., 2023). Direct pharmacological activation of V-ATPase remains challenging; however, late endolysosomal function can be modulated through related pathways, such as activation of the endolysosomal Ca^2 +^ channel TRPML1 (Pollmanns et al., 2022). For example, ML-SA1 activates TRPML1 and promotes late autophagy steps, thereby facilitating the clearance of α-Syn aggregates in cellular models, but this effect should not be described as direct V-ATPase activation (Pollmanns et al., 2022; Xia et al., 2020; Zhang S. et al., 2025). Stabilizing lysosomal membranes is another experimental approach, because α-Syn oligomers or fibrils can disrupt endolysosomal membrane integrity and promote cathepsin leakage (Crunkhorn, 2016; Dai et al., 2024; Dilsizoglu Senol et al., 2021; Freeman et al., 2013; Nylandsted et al., 2004). Hsp70 itself can reduce α-Syn aggregation and toxicity and has been shown to inhibit LMP, suggesting that chaperone-based modulation may protect lysosomal integrity; however, specific Hsp70-inducing drugs should be discussed cautiously unless direct PD or α-Syn evidence is available (Kirkegaard et al., 2010; Klucken et al., 2004; Nylandsted et al., 2004). Lipid-modulating approaches may also be relevant, because lysosomal and endolysosomal membrane composition influences α-Syn handling. For example, 2-hydroxypropyl-β-cyclodextrin improved PD-related phenotypes in patient-derived neuronal cultures and brain organoids, although its relationship to lysosomal membrane stabilization and α-Syn clearance requires careful interpretation (Jarazo et al., 2022). Collectively, lysosome-directed therapies aim to restore enzyme activity, improve endolysosomal trafficking and acidification, preserve membrane integrity, and support α-Syn clearance. Nevertheless, most strategies remain at preclinical or early clinical stages, and future studies must establish target engagement, CNS delivery, patient selection, and meaningful disease-modifying effects in human PD.

### 4.2 Autophagy inducers and modulators

Macroautophagy and chaperone-mediated autophagy (CMA) are complementary lysosome-dependent pathways involved in α-Syn proteostasis: macroautophagy is more relevant to larger aggregates and damaged organelles, whereas CMA preferentially handles soluble and modified α-Syn species (Cuervo et al., 2004; Martinez-Vicente et al., 2008; Webb et al., 2003). Pharmacological modulation of autophagy has therefore been explored as a potential therapeutic approach in PD models, but translation requires careful control of pathway specificity, disease stage, and lysosomal degradative capacity (Khan et al., 2023; Spencer et al., 2009; Xilouri et al., 2013b). Rapamycin and related mTORC1 inhibitors activate macroautophagy by suppressing mTORC1, a major negative regulator of autophagy initiation (Hara et al., 2002; Khan et al., 2023). In experimental PD models, rapamycin or mTORC1 inhibition has been reported to protect dopaminergic neurons, improve some behavioral outcomes, or reduce α-Syn-related pathology in a model-dependent manner; however, broad mTORC1 inhibition may also affect protein synthesis, metabolism, immunity, and neuronal survival pathways (Khan et al., 2023; Liu et al., 2013). These limitations have encouraged interest in mTOR-independent or pathway-selective autophagy enhancers that may increase clearance without globally suppressing mTORC1 signaling (Sarkar et al., 2005; Sarkar et al., 2007; Williams et al., 2008). Among mTOR-independent or partially mTOR-independent approaches, trehalose has been reported to enhance autophagy and accelerate clearance of α-Syn in experimental systems, while lithium can induce autophagy through inositol monophosphatase inhibition and reduced IP3 signaling (Sarkar et al., 2005; Sarkar et al., 2007). Metformin may also modulate autophagy and mitochondrial metabolism, but its mechanisms in PD models are pleiotropic and should not be reduced to a single pathway (Agostini et al., 2021; Gopar-Cuevas et al., 2023). Trehalose, a disaccharide autophagy enhancer, has been shown to accelerate α-Syn clearance in cellular systems and to improve behavioral or neurochemical outcomes in α-Syn-based animal models, but its limited bioavailability and blood–brain barrier penetration remain important translational obstacles (He et al., 2016; Sarkar et al., 2007; Yoon et al., 2017). CMA enhancement is another more selective approach because CMA preferentially degrades soluble α-Syn species and may help prevent their transition into aggregation-prone intermediates (Cuervo et al., 2004; Martinez-Vicente et al., 2008; Xilouri et al., 2013b). Instead of QBP1, the most direct evidence for CMA enhancement in α-Syn models comes from increasing LAMP2A expression or activating CMA-related transcriptional programs (Xilouri et al., 2013b). LAMP2A overexpression enhances CMA activity and mitigates α-Syn-induced neurodegeneration in vivo, while NRF2-dependent LAMP2A regulation and compounds such as apigenin provide additional experimental routes for CMA activation (Huang et al., 2025; Pajares et al., 2018). Overall, autophagy modulators aim to improve the balance between α-Syn production and clearance, but their therapeutic value will depend on whether they can restore functional flux without overloading lysosomes, disrupting essential mTOR signaling, or producing unacceptable systemic effects.

### 4.3 Interventions targeting α-Syn aggregation and propagation

Targeting α-Syn aggregation and intercellular propagation is a rational therapeutic strategy, because these processes may contribute to pathological amplification and regional spread in PD (Arias-Carrión et al., 2025; Desplats et al., 2009; Fields et al., 2019; Luk et al., 2009; Luk et al., 2012). Active and passive immunotherapy approaches have been developed to bind extracellular α-Syn species, reduce seed availability, and potentially limit cell-to-cell transmission (Lang et al., 2022; Pagano et al., 2022; Volc et al., 2020). Active immunization strategies such as PD01A aim to elicit endogenous anti-α-Syn antibody responses, whereas passive immunization with monoclonal antibodies such as prasinezumab or cinpanemab aims to bind extracellular α-Syn species and interfere with propagation-related mechanisms (Lang et al., 2022; Murakami et al., 2023; Pagano et al., 2022; Polissidis et al., 2020; Volc et al., 2020). More recently, UB-312 has also shown safety, immunogenicity, and preliminary target-engagement signals in early clinical studies (Yu et al., 2022). In the PASADENA phase 2 trial, prasinezumab did not show a meaningful effect on global or imaging measures of PD progression, although subsequent analyses suggested possible motor-symptom signals in selected or rapidly progressing subgroups; these findings remain exploratory and require confirmation (Pagano et al., 2022). Small-molecule anti-aggregation strategies, including oligomer modulators such as anle138b and α-Syn misfolding inhibitors such as NPT200-11, aim to reduce toxic oligomer formation, fibrillization, or α-Syn-mediated pathology through compound-specific mechanisms (Peña-Díaz et al., 2023; Price et al., 2018; Vidović and Rikalovic, 2022; Wagner et al., 2013). Anle138b reduced α-Syn oligomer accumulation and improved disease-related phenotypes in preclinical synucleinopathy models, and phase 1 studies have primarily evaluated its safety, tolerability, and pharmacokinetics rather than clinical efficacy (Levin et al., 2022; Wagner et al., 2013). Gene silencing technologies, including antisense oligonucleotides (ASOs) and small interfering RNAs (siRNAs), target *SNCA* mRNA to reduce α-Syn protein synthesis (Cogan and Brice, 2025; Cole et al., 2021). Preclinical *SNCA*-targeting ASOs have shown broad CNS distribution and α-Syn lowering in rodent and non-human primate studies, supporting clinical development, but long-term safety and appropriate dosing require careful evaluation (Alarcón-Arís et al., 2020; Cole et al., 2021). Together, these approaches target different levels of α-Syn biology, including extracellular seeding, aggregate formation, and α-Syn production. However, clinical translation remains challenging because target engagement in the human brain, optimal disease stage, patient selection, and long-term safety have not yet been fully established.

## 5 Discussion

Parkinson’s disease is increasingly understood as a multifactorial neurodegenerative disorder in which abnormal protein aggregation, impaired intracellular degradation, mitochondrial dysfunction, lipid metabolic disturbance, and neuroinflammation interact to drive progressive neuronal injury (Alecu and Bennett, 2019; Ebrahimi-Fakhari et al., 2012; Picca et al., 2021). Within this complex pathological network, the α-Syn-lysosome axis represents a particularly important mechanistic framework because it links two central features of PD pathogenesis: the accumulation of misfolded α-Syn and the failure of lysosome-dependent proteolytic clearance (Dehay et al., 2013; Navarro-Romero et al., 2020; Wildburger et al., 2020). The present review highlights that α-Syn pathology is not merely a downstream consequence of neuronal degeneration, but may actively participate in a self-amplifying cycle in which toxic α-Syn species impair lysosomal function, while lysosomal dysfunction further accelerates α-Syn accumulation, aggregation, and propagation (Bellomo et al., 2020; Hoffmann et al., 2019; Mazzulli et al., 2011; Mazzulli et al., 2016a; Wildburger et al., 2020). This bidirectional relationship provides a coherent explanation for why disturbances in proteostasis are closely associated with the progressive nature of PD and why restoring lysosomal and autophagic capacity has become an important focus of mechanism-based therapeutic research.

A key implication of this review is that lysosomal dysfunction in PD should not be regarded as a single pathological event, but rather as a multidimensional process involving enzymatic insufficiency, defective acidification, impaired membrane integrity, and altered vesicular trafficking (Colacurcio and Nixon, 2016; Dai et al., 2024; Dehay et al., 2013; Mazzulli et al., 2016a; Navarro-Romero et al., 2020). Among these mechanisms, GBA1-associated glucocerebrosidase deficiency provides one of the strongest genetic and mechanistic links between lysosomal dysfunction and α-Syn pathology (Alcalay et al., 2015; Mazzulli et al., 2011; Stojkovska et al., 2018; Wildburger et al., 2020). Reduced GCase activity leads to glycosphingolipid accumulation, impaired lysosomal homeostasis, and decreased α-Syn degradation, whereas accumulated α-Syn can further inhibit GCase trafficking and activity, forming a pathogenic feedback loop (Mazzulli et al., 2011; Mazzulli et al., 2016b; Wildburger et al., 2020; Yap et al., 2013). Similarly, V-ATPase dysfunction and lysosomal alkalization reduce the activity of lysosomal hydrolases and compromise the degradative environment required for α-Syn clearance (Colacurcio and Nixon, 2016; Song et al., 2020; Zhang S. et al., 2025). Lysosomal membrane permeabilization further extends this pathological cascade by allowing cathepsins to leak into the cytoplasm, thereby promoting lysosome-dependent cell death, oxidative stress, and inflammatory signaling (Codolo et al., 2013; Freeman et al., 2013; Qiao et al., 2016; Roberg et al., 2002; Wang et al., 2018). These findings suggest that different forms of lysosomal injury may converge on a common outcome: the weakening of neuronal proteostasis and the amplification of α-Syn toxicity.

Autophagy impairment constitutes another essential component of the α-Syn-lysosome axis (Arotcarena et al., 2019; Nechushtai et al., 2023; Xilouri et al., 2016). Macroautophagy, chaperone-mediated autophagy, and mitophagy are not isolated degradation systems, but functionally interconnected processes that maintain neuronal homeostasis (Fleming et al., 2022; Lizama and Chu, 2021; Wang et al., 2016; Wu et al., 2015). In PD, blockade of autophagic flux may occur at multiple stages, including autophagosome formation, cargo recognition, vesicular transport, and autophagosome-lysosome fusion (Lei et al., 2019; Sarkar et al., 2021; Tang et al., 2021; Watanabe et al., 2012; Winslow and Rubinsztein, 2011; Winslow et al., 2010). α-Syn itself can interfere with autophagic machinery by disrupting SNARE-dependent fusion events and by altering selective autophagy pathways involving receptors such as p62/SQSTM1 (Sarkar et al., 2021; Tang et al., 2021; Xu et al., 2025). In parallel, CMA dysfunction is particularly relevant because soluble α-Syn is a direct substrate of this pathway (Alvarez-Erviti et al., 2010; Cuervo et al., 2004; Sala et al., 2016; Vogiatzi et al., 2008). Pathogenic α-Syn mutations or dopamine-modified α-Syn can bind to LAMP2A but fail to undergo efficient translocation, thereby blocking CMA and promoting further cytosolic accumulation of α-Syn (Alvarez-Erviti et al., 2010; Cuervo et al., 2004; Martinez-Vicente et al., 2008). These observations support the view that autophagy failure is both a cause and a consequence of α-Syn accumulation, reinforcing the need to target autophagic flux rather than focusing solely on α-Syn aggregation.

The interaction between autophagy impairment and mitochondrial dysfunction further broadens the pathogenic significance of the α-Syn-lysosome axis (Lin et al., 2019; Minakaki et al., 2020; Zhang et al., 2018). Dopaminergic neurons are highly dependent on mitochondrial function because of their extensive axonal arborization, high energetic demand, and vulnerability to oxidative stress (Pacelli et al., 2015; Pissadaki and Bolam, 2013; Surmeier, 2018). Defective mitophagy caused by mutations or dysfunction in genes such as *PINK1*, *PRKN*, and *LRRK2* leads to the accumulation of damaged mitochondria, increased reactive oxygen species production, and reduced ATP generation (Bonello et al., 2019; Eldeeb et al., 2022; Narendra et al., 2008; Narendra et al., 2010; Singh and Ganley, 2021). These changes can promote oxidative modification and aggregation of α-Syn, while α-Syn aggregates further impair lysosomal function and mitophagic clearance (Parihar et al., 2009; Paxinou et al., 2001; Shaltouki et al., 2018; Thorne and Tumbarello, 2022; Won et al., 2022). Therefore, mitochondrial dysfunction and lysosomal-autophagic impairment appear to form an interconnected degenerative loop rather than two independent pathways. This concept is consistent with the current mainstream understanding that PD pathogenesis involves the convergence of proteinopathy, organelle quality-control failure, and cellular stress responses.

From a therapeutic perspective, targeting the α-Syn-lysosome axis offers several attractive opportunities, but also presents important translational challenges (Dehay et al., 2015; Grosso Jasutkar et al., 2022; Lang and Espay, 2018). Strategies aimed at enhancing lysosomal function, including GCase activation, lysosomal re-acidification, stabilization of lysosomal membranes, and modulation of lysosomal biogenesis, are conceptually well aligned with the pathogenic mechanisms discussed in this review. Autophagy modulators, including mTORC1-dependent and mTOR-independent approaches, may promote α-Syn clearance in experimental models, whereas CMA-enhancing strategies may be particularly relevant for soluble α-Syn species before they enter aggregation-prone states (Kinet and Dehay, 2023; Li et al., 2011; Moors et al., 2017; Xilouri et al., 2013a; Xilouri et al., 2013b; Zhang et al., 2021). In addition, immunotherapies, aggregation inhibitors, and SNCA-lowering strategies directly reduce α-Syn burden by targeting extracellular propagation, oligomer formation, or α-Syn synthesis (Dehay et al., 2015; Menon et al., 2022; Uehara et al., 2019). However, the clinical translation of these approaches remains complex (Lang and Espay, 2018). For example, clinical trials targeting α-Syn have yielded heterogeneous results, suggesting that patient selection, disease stage, target engagement, and biomarker-guided outcome assessment are critical for future therapeutic success (Lang et al., 2022; Mollenhauer et al., 2017; Pagano et al., 2021; Pagano et al., 2022; Pagano et al., 2024).

An important challenge in this field is that many mechanistic findings are still derived from cellular and animal models, whereas robust validation in human PD tissues and longitudinal clinical cohorts remains limited. Although preclinical models are indispensable for dissecting molecular mechanisms, they cannot fully reproduce the long prodromal phase, regional vulnerability, aging-related changes, genetic heterogeneity, and mixed pathologies observed in human PD (Dawson and Dawson, 2025; Day and Mullin, 2021; Dovonou et al., 2023; Klæstrup et al., 2022; Walker and Attems, 2024; Yamakado and Takahashi, 2024). Moreover, the α-Syn-lysosome axis may differ among sporadic PD, GBA1-associated PD, LRRK2-associated PD, and other synucleinopathies (Skrahin et al., 2024; Smith et al., 2022). Therefore, future studies should avoid treating PD as a biologically uniform disease and should instead adopt a stratified research framework based on genetic background, molecular biomarkers, α-Syn burden, lysosomal activity, and clinical phenotype. Such an approach may help identify which patient subgroups are most likely to benefit from GCase enhancement, autophagy modulation, α-Syn immunotherapy, or combination treatment.

Future research should also place greater emphasis on the development of reliable biomarkers that reflect α-Syn aggregation, lysosomal dysfunction, and autophagic flux *in vivo*. Peripheral α-Syn detection, lysosomal enzyme activity assays, extracellular vesicle profiling, cerebrospinal fluid biomarkers, imaging approaches, and multi-omics analyses may provide complementary information for disease diagnosis, progression monitoring, and therapeutic evaluation. In addition, human-derived models, including induced pluripotent stem cell-derived dopaminergic neurons, organoids, and striatal-midbrain assembloids, may provide more physiologically relevant platforms for studying α-Syn propagation and testing therapeutic candidates. Combining these models with single-cell sequencing, spatial proteomics, and real-time autophagic flux assays may further clarify how neuron-glia interactions, lipid metabolism, mitochondrial stress, and lysosomal impairment jointly shape PD progression.

Overall, the α-Syn-lysosome axis provides an integrated conceptual framework for understanding the convergence of α-Syn aggregation, lysosomal dysfunction, autophagy impairment, and mitochondrial injury in PD. Current evidence supports the view that restoring proteostatic balance is a rational therapeutic direction, particularly if interventions can be applied before irreversible neuronal loss occurs. Nevertheless, future therapeutic development should move beyond single-target approaches and consider the dynamic, bidirectional, and patient-specific nature of this axis. A combination of mechanism-based drug development, biomarker-guided patient stratification, and clinically relevant human models will be essential for translating advances in α-Syn and lysosomal biology into effective interventions for PD.
